# Assessment of Immunization to Hepatitis B Vaccine among Children under Five Years in Rural Areas of Taiz, Yemen

**DOI:** 10.1155/2017/2131627

**Published:** 2017-03-06

**Authors:** Fuad A. A. Alssamei, Najla A. Al-Sonboli, Fawzi A. Alkumaim, Nader S. Alsayaad, Mohammed S. Al-Ahdal, Tarig B. Higazi, Atif A. Elagib

**Affiliations:** ^1^Microbiology and Immunology Departments, Military Hospital, Sana'a, Yemen; ^2^Pediatrics Department, Faculty of Medicine and Health Sciences, Sana'a University, Sana'a, Yemen; ^3^Pediatrics Department, Al-Sabeen Maternity and Child Hospital, Sana'a, Yemen; ^4^Virology and Serology Department, Military Hospital, Sana'a, Yemen; ^5^Department of Biological Sciences, Ohio University Zanesville, Zanesville, OH, USA; ^6^National Center for Research, Khartoum, Sudan

## Abstract

*Background*. Hepatitis B virus (HBV) infection poses a major health problem worldwide. approximately 1 million deaths annually due to cirrhosis and hepatocellular carcinoma.* Objectives*. This study was conducted to determine the coverage rate of HBV vaccine and assess the vaccine protective response among children under five years old in rural areas of Yemen.* Methods*. A cross-sectional study was conducted from January to December 2015 in four districts of countryside Yemen. The target population was children aged from 6 to 59 months. 227 children were enrolled in the study. Questionnaire was used to collect of data. Serum samples were tested for anti-HBs antibodies by enzyme linked immunosorbent assay (ELISA). Anti-HBs level ≥ 10 IU/L was considered a protective response to the vaccine.* Results*. The coverage rate of HBV vaccine among children was 87.3%. A total of 143 (72.2%) children responded to the vaccine with anti-HBs level ≥ 10 IU/L, while 55 (27.8%) of the children had nonprotective anti-HBs levels of <10 IU/L (*P* = 0.003).* Conclusion*. This study revealed a good coverage rate of HBV vaccine in rural areas but the protective rate against HBV infection was moderate. A considerable proportion of vaccinated children should be considered for either revaccination or booster doses.

## 1. Introduction

A major cause of morbidity and mortality in children are infectious diseases, including hepatitis B [1]. Hepatitis B virus (HBV) infection remains a global challenge, with one-third of the world's population having serological evidence of current or previous infection. Around 400 million people worldwide are chronically infected with HBV that leads to approximately 1 million deaths annually due to cirrhosis and hepatocellular carcinoma [2]. Primary prevention by immunization remains the most effective way to control the spread of HBV especially in developing country [3]. However, it is estimated that every year at least 27 million children worldwide do not receive the basic package of immunizations. About 25% of children under five years' mortality is due to infectious diseases preventable by vaccine [4, 5]. Previous surveys carried out in Yemen showed high prevalence of hepatitis B surface antigen (HBsAg) ranging from 8 to 20% [6] and up to 50% in the capital Sana'a [7]. Other studies in Yemen reported HBsAg overall prevalence of 10.5% in Sana'a, 4.8% in Aden, 5.6% in Hajah, 26.3% in Soqotra [8], and 16.9% in Taiz [9] governorate during the period between 2000 and 2005. It has been reported that chronic infection generally develops among 90% of newborns, 29–40% of children (1–5 years old), and 5–10% of adults [10].

With the availability of HBV vaccine since 1982, the decline in the incidence of HBV infection and associated morbidity and mortality has been reported in Taiwan and the United States [11, 12]. In 1998, the World Health Organization (WHO) recommended the inclusion of HBV vaccine in the national immunization program of Yemen particularly among neonates, where vertical transmission is common, regardless of the prevalence of circulating HBsAg [13]. Despite a remarkable success in immunization coverage in Yemen, there are still areas of low coverage and a gap still exists between urban and rural areas. Nearly 13.5 million (54%) Yemenite live below the poverty line and the majority of them (66–87%) live in rural areas [14, 15]. Factors associated with decreased immune response to HBV vaccine include increasing age, gender, obesity, nutritional status, smoking, and genetic factors [16, 17]. Poverty, socioeconomic status, lack of education, and weak health systems in rural areas in Yemen are interrelated factors that influence growth of children, which in turn affect their immune system [14] and response to vaccines.

In general, three doses of HBV vaccine provide a safe level of protection in 95% of healthy infants and healthy children. The primary immune response to HBV vaccine decreases with increasing age, especially after 40 years old as it declines to 90% [18]. Studies in Yemen have demonstrated that immunogenicity of HBV vaccine in healthy infants and children varies from 54.8% to 83.5% [13, 19, 20] as evident by antibodies to HB surface antigen (anti-HBs) response of ≥10 IU/L to pentavalent or a single vaccine. However, these studies were mainly conducted in urban [13, 20] or a mixed sample of both rural and urban areas [19]. A recent study conducted in Taiz found low routine vaccination coverage rate (69.5%) among children aged 12–23 months, with a highest coverage rate of 82.6% for HBV vaccine [21]. More than 130 million children are born each year worldwide and need to be immunized. Over 27 million children, living mainly in rural communities, are not reached by routine immunization services and significant variations in coverage rate are found between and within regions and countries [22].

In Yemen, health care facilities are mainly concentrated in urban centers and developed governorates leading to 80% health coverage in urban areas in comparison to only 25% coverage in the rural areas [14]. Our study assesses an outcome of these gaps by evaluating the coverage rate of HBV vaccine and the protective immune response to HBV vaccine among children under five years old vaccinated with the three doses of HBV vaccine who live in rural areas around Taiz, Yemen.

## 2. Materials and Methods

### 2.1. Study Population

This cross-sectional study was conducted from January to December 2015 at four rural districts including 11 villages in Taiz governorate, Yemen (Table 1). These districts represent about one-third of rural districts in Taiz governorate and the 11 villages are located within about 60 kilometers from the city center of Taiz. Districts and villages selection was based on geographical location and population size per village drawn from the latest population census of 2004. The target population was children aged from 6 to 59 months of a total number of 1789 individuals in the study area. All 228 children aged 6–59 months registered in the rural health units within the study area were enrolled in the study (Table 1). Information about vaccination status according to the last dose of HBV vaccine, sex, and age at the time of the study has been obtained through face to face interview with the children guardians. Ethical approval for this study was obtained from the ministry of public health and population in Yemen. Informed consent was obtained from the parent or guardian of the children enrolled in the study following explanation of the goal of the study.

### 2.2. Measurement of HBV Markers

Two milliliters of venous blood samples was collected from 228 children enrolled in the study based on the criteria listed above and sera were separated and frozen at −20°C until testing. Natural immunity to HBV infection was first excluded by screening for total anti-HB core antibodies as previously described [13]. One child who had anti-HB core antibodies was excluded from study. Rest of the sera were then tested for anti-HBs using recombinant Hepatitis B virus surface antigen (rHBs Ag) found in the sandwich ELISA AxSYM AUSAB kit following the manufacturer protocol (Abbott, Germany). Anti- HBs antibody concentration was expressed in international units per liter (IU/L). The protective immune response was defined as anti HBs antibodies level of ≥10 IU/L [23, 24].

### 2.3. Statistical Analysis

The data was analyzed using version 17 of the Statistical Package of Social Science (SPSS) (SPSS Inc., Chicago, IL, USA). For the qualitative data (frequency and proportion), the Chi-Square test was used statistically to compare observed data with expected data. All differences were considered statistically significant when the Probability values (*P* value) were < 0.05. Correlation coefficient and differences between anti-HBs levels were measured using the Spearman's and one way ANOVA tests of the SPSS package.

## 3. Results

**Figure 1:**
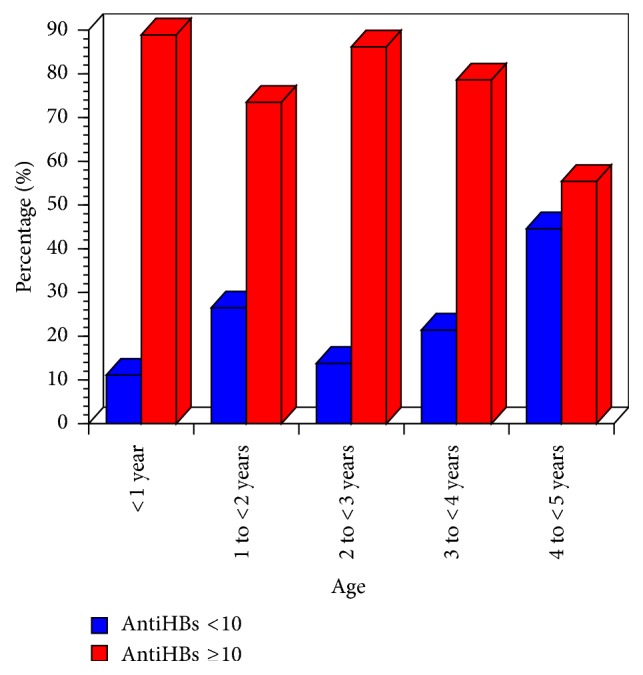
Comparison of anti-HBs antibody levels in vaccinated 6–59-month children according to age groups.

A total of 227 children with mean age of 36.44 ± 18.5 SD months, 108 (47.6%) males and 119 (52.4%) females, were included in this study. One hundred and ninety-eight children were vaccinated (87.3%) and 29 (12.8%) unvaccinated indicating 87.3% coverage rate of HBV vaccine. HBV vaccine coverage was 88% and 86.6% for males and females, respectively (Table 2). In this study, 143 (72.2%) of 198 children showed anti-HBs protective level of ≥10 IU/L, while 55 (27.8%) showed nonprotective anti-HBs titer levels of <10 IU/L. Protective anti-HBs levels were slightly higher in females (75.7%) than males (68.4%) (Table 3) but this difference was not statistically significant (*P* = 0.251). Anti-HBs antibody levels were then compared in various age groups (<1 year, 1 to >2 years, 2 to <3 years, 3-to <4 years, and 4 to <5 years old). Protective responses to HBV vaccine as measured by levels of anti-HBs differed significantly between age groups (*P* = 0.014) (Table 4) with 88.9% protective rate in infant children less than one year and 55.4% rate for children aged in the 4- to <5-year-old age group (Figure 1). Anti-HBs levels were found to decrease with the increasing age (correlation coefficient = −0.306) with the second year group response being less than the third and fourth year groups (Table 4) (Figure 1).

## 4. Discussion

This study showed relatively high HBV vaccination coverage rate of 87.3% in rural area around Taiz, Yemen. These finding are similar to our recent study conducted on malnourished children in Yemen [19] and higher than the 70% rate reported from Sana'a city in 2011 [13]. This higher HBV vaccine coverage rate might be attributed to increased response to the ministry of public health and population awareness and educational programs and increased efficiency of vaccination campaigns. The national health status in Yemen has been in steady growth recently due to the concerted efforts to educate the public about the importance of immunization in the fight against infectious childhood diseases in minimizing mortality rate. However, our study showed lower coverage compared to endemic developed [25] and developing [26] countries, where HBV vaccine coverage rates among children ranged from 90 to 98%.

The present study revealed 72.2% protective rate (anti-HBs ≥ 10 IU/L) for vaccinated healthy children in rural areas of Taiz. This HBV vaccine seroprotective rate was higher than the rate reported in children <1–10 years old by Al Shamahy and coworkers in Sana'a 5 years ago [13]. However, it was lower than that reported by Sallam and coworkers from Sana'a in 2005 [20]. In the same study, however, Sallam and coworkers noted significantly lower HBV antibody level among children with low economic status. These variations in effectiveness of vaccine may be as a result of differences in socioeconomic status, health care program, and ethnic differences between populations. Lower levels of anti-HBs antibodies could also be related to existing problems with the cold chain of vaccines in rural areas that lead to decreased efficacy of the pentavalent vaccine. Lower responses to HBV vaccine in low socioeconomic areas have been reported in Taiwan [27] and significant correlation between nutritional status and the response to HBV vaccination has been reported in Senegal and Cameron [28]. In addition, Losonsky and coworkers reported association between low weight and poor weight gain in the first 6 months of life with decreased immunogenicity after three doses of HBV vaccine in the United States [29].

Our study also showed slightly higher protective rate of anti-HBs antibody in females 78 (75.7%) compared to males (68.4%). Similar gender-based HBV vaccine protective rates were reported in previous studies conducted in Yemen [19, 20] and in China [30]. This gender-based variation may be possibly due to the physiological and behavioural differences between the gender which plays important role in immune response [31, 32]. In particular, females mount higher innate and adaptive immune responses to pathogen challenge than males do. Fish and coworkers found constitutively higher levels of circulating T cells in females compared with males, which probably contribute to gender-differential nonspecific effects (NSE) of vaccines [33]. In addition, Klein and coworkers [34] reported more robust innate immune responses in females to the yellow fever vaccine as well as higher antibody responses in females to the influenza vaccine, combined measles mumps rubella (MMR) vaccine, and hepatitis A and B vaccines [34]. Moreover, mice studies showed that males generally produce more proinflammatory cytokines, such as IL-6 and TNF, while females produce more of the anti-inflammatory cytokines [35]. These data support the gender-based differences in response to HBV vaccination reported in this study.

This study showed significant differences and negative correlation in HBV vaccine protection rate between age groups (<1–5 years), with highest protective rate in the less than one year age group. The low correlation value was due to the weaker protective response of the one to <2 years old group compared to the older age group. This finding was supported by previous reports of declined HBV vaccine protective level with age in Yemen [13, 19]. Similar association between older age and lower level of HBV vaccine protection rate has been reported by studies conducted in Saudi Arabia [36], Europe [37], and China [38, 39]. A well-described age-related modulation of the immune system is the decline of de novo generation of T and B cells. In addition, the accumulation of memory cells and loss of diversity in antigen specificities caused by a lifetime of exposure to pathogens have also been described [40].

Our study has been limited by sample size, geographic area, and difficulty to interpret associations. Additional studies with larger sample size and more representative areas are needed to verify the role of factors such as age, gender, race, site of injection, nutritional status, and vaccine brand on responses to HBV vaccine in Yemen.

## 5. Conclusions

This study showed good coverage and moderate protective rate against HBV in rural areas around Taiz. Further studies are recommended to provide better estimates of HBV vaccine coverage and rate of the vaccine protection among Yemeni children and to detect the factors that affect the vaccine protective rates. Such information is important for policy makers for planning strategies to improve these rates. The study also estimated proportion of vaccinated children in rural areas of Taiz, Yemen who should be considered for either revaccination or booster doses.

## Figures and Tables

**Table 1:** Number and distribution of 6–59-month-old children study population among districts and villages of rural Taiz, Yemen.

District	Villages	Number of children
Sama	Aldar	60
	Alhub	31
	Almanagl	24

*Total*	*3*	*115*

Al Ma'afer	Alarida	13
	Alsheib	21

*Total*	*2*	*34*

Al Mawasit	Almanhi	13
	Almarafed	16
	Hagafer	14

*Total*	*3*	*43*

Almsrakh	Aldera	10
	Hugmah	18
	Alkehaf	7

*Total*	*3*	*35*

*Grand total*	*11*	*227*

**Table 2:** The coverage rate of hepatitis B vaccine (HBV) according to gender.

Status	Males		Females		Total	
	Number	(%)	Number	(%)	Number	(%)
Vaccinated	95	(88%)	103	(86.6%)	198	(87.2%)
Nonvaccinated	13	(12%)	16	(13.4%)	29	(12.8%)

Total	108	(100%)	119	(100%)	227	(100%)

**Table 3:** Gender-based Immune response to HBV vaccine among 6–59-month-old vaccinated children in rural areas of Taiz, Yemen.

Anti-HBs titer (IU/L)	Males		Females		Total	
	Number	(%)	Number	(%)	Number	(%)
<10	30	(31.6%)	25	(24.3%)	55	(27.8%)
10–100	26	(27.4%)	25	(24.3%)	51	(25.8%)
101–999	33	(34.7%)	38	(36.9%)	71	(35.9%)
≥1000	6	(6.3%)	15	(14.6%)	21	(10.6%)

Total	95	(100%)	103	(100%)	198	(100%)

**Table 4:** Comparison of the geometric mean of protective Anti-HBs levels (≥10 IU/L) in HBV vaccinated children by age groups by one-way ANOVA.

Age (years)	No	Anti-HBs geometric mean	*F*	*P* value
<1	27	189.3004	3.229	0.014
1 to <2	49	60.0875		
2 to <3	29	99.9618		
3 to <4	28	81.4639		
4 to <5	65	21.2379		

Total	198	56.1740		
